# New Rimocidin/CE-108 Derivatives Obtained by a Crotonyl-CoA Carboxylase/Reductase Gene Disruption in *Streptomyces diastaticus* var. 108: Substrates for the Polyene Carboxamide Synthase PcsA

**DOI:** 10.1371/journal.pone.0135891

**Published:** 2015-08-18

**Authors:** Leticia Escudero, Mahmoud Al-Refai, Cristina Nieto, Hartmut Laatsch, Francisco Malpartida, Elena M. Seco

**Affiliations:** 1 Centro Nacional de Biotecnología (CNB-CSIC), Campus de la Universidad Autónoma de Madrid, Cantoblanco, 28049, Madrid, Spain; 2 Department of Organic and Biomolecular Chemistry, University of Göttingen, Tammannstrasse 2, D-37077, Göttingen, Germany; University of Freiburg, GERMANY

## Abstract

The *rimJ* gene, which codes for a crotonyl-CoA carboxylase/reductase, lies within the biosynthetic gene cluster for two polyketides belonging to the polyene macrolide group (CE-108 and rimocidin) produced by *Streptomyces diastaticus* var. 108. Disruption of *rimJ* by insertional inactivation gave rise to a recombinant strain overproducing new polyene derivatives besides the parental CE-108 (**2a**) and rimocidin (**4a**). The structure elucidation of one of them, CE-108D (**3a**), confirmed the incorporation of an alternative extender unit for elongation step 13. Other compounds were also overproduced in the fermentation broth of *rimJ* disruptant. The new compounds are *in vivo* substrates for the previously described polyene carboxamide synthase PcsA. The *rimJ* disruptant strain, constitutively expressing the *pcsA* gene, allowed the overproduction of CE-108E (**3b**), the corresponding carboxamide derivative of CE-108D (**3a**), with improved pharmacological properties.

## Introduction

The polyene-macrolides are a group of polyketides that are commercially important because of their antifungal properties. This group includes well-known drugs such as amphotericin B (**1**), pimaricin, nystatin, **4a**, and candicidin. They consist of a macrolactone ring containing several conjugated double bonds, which are in part responsible for the physical and chemical properties of these compounds (strong light sensitivity and low solubility in water). A sugar moiety (typically mycosamine) and a free carboxyl group are usually found on the macrocycle. The biological activity of these polyenes is rather specific for fungi, due to their preferred affinity toward ergosterol-containing membranes (fungal membranes and the membranes of some parasites such as *Trypanosoma*, *Leishmania*, etc.) rather than cholesterol-containing membranes. This interaction seems to affect some physico-chemical properties of the membranes, leading to changes on their ionic permeability, pore formation, loss of ions and thus cell death. In contrast to other antifungal drugs, the rate of appearance of resistant forms of the target microorganisms after treatment with polyenes is very low.

**Fig 1:**
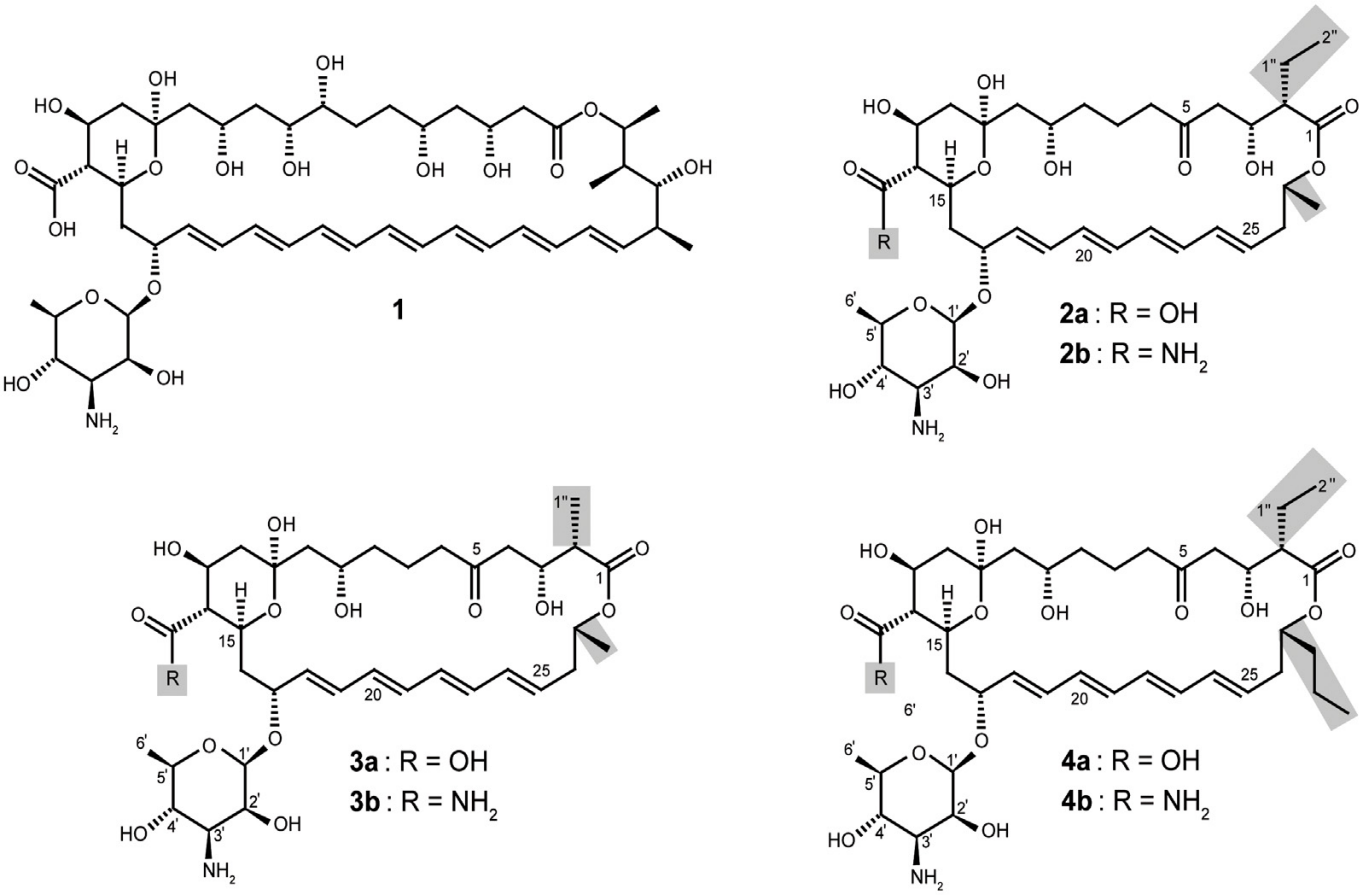
Chemical structures of polyenes cited in the text. **1**: Amphotericin B; **2a**: CE-108; **2b**: CE-108B; **3a**: CE-108D; **3b**: CE-108E; **4a**: Rimocidin; **4b**: Rimocidin B. Note that the depicted stereochemistry of **2a**, **2b**, **3a**, **3b** and **4b** was deduced from the known stereoschemistry of **4a** and has not been experimentally established.

Undoubtedly the most important drawback for the clinical use of some polyenes like amphotericin B (**1**) (Fig 1) are the undesirable side effects during treatment of systemic fungal infections, particularly nephrotoxicity and hepatotoxicity. These toxicities seem to be associated with the interactions between **1** and cell membranes [1]. The need for new alternative antifungals with improved pharmacological properties has given rise to semi-synthetic derivatives of **1** designed to reduce the undesirable side effects while retaining antifungal activity. These attempts yielded new derivatives with good prospects as drugs and, more importantly, have increased knowledge of structure-activity relationships. Thus, exchanging several functional groups of **1**, such as the carboxyl group of the macrolactone ring and/or the amino group of the mycosamine sugar or the polyol chain, seems to be crucial for the improvement of pharmacological properties (lower toxicity and increased antifungal activity and water solubility) [2–4]. Semisynthetic derivatives of other polyene macrolides have been developed with similar results [5,6]. Polyenes, like all macrolides, are produced through the action of type I modular polyketide synthases (PKSs). These large multifunctional enzymes consist of distinct domains, each of which catalyzes one non-iterative polyketide chain elongation step [7]. The availability of biosynthetic genes for several polyene macrolide pathways [8] has provided additional tools for *in vivo* biosynthesis of new chemical entities. In this way, new derivatives were successfully obtained by genetic engineering of polyene producer strains [9–18]. Due to the chemical complexity of the polyene macrolides, the biosynthetic route seemed to be a highly promising alternative for affording chemical modification of the macrolactone ring and thus generation of derivatives with improved pharmacological properties.

We have previously characterized a chromosomal region of *Streptomyces diastaticus* var. 108 [19] encoding the biosynthetic machinery for two related tetraenes: **4a** and **2a** (Fig 1). Based on their chemical structures, we proposed a model for the biosynthetic pathway [20]. The incorporation of a malonyl-CoA or ethylmalonyl-CoA starter unit determines the formation of **2a** and **4a**, respectively. RimA (module 0), the loading PKS, exhibits the domain structure carboxylic acid:CoA ligase-ACP-KS^S^-AT-ACP, in which the KS^S^ domain (ketosynthase with a serine residue in place of the conserved active site cysteine) would not catalyze a condensation reaction, but rather select and possibly decarboxylate the starter unit, before transferring it to the C-terminal ACP. This peculiar KS^S^ is also present in other polyene loading PKSs [21–23]. It is known that KS domains are converted to potent decarboxylases when the active site cysteine is replaced with glutamine [24,25] so this domain could function as a decarboxylase that acts on malonyl-CoA or ethylmalonyl-CoA to generate acetyl-CoA and butyryl-CoA starter units, respectively. Site-directed mutagenesis of the KS^S^ domain of NysA, the loading PKS for nystatin biosynthesis, surprising suggested that the conserved serine residue S413 may be important for the decarboxylation of the malonyl-CoA starter unit, rater than S170 that sits in place of the active site cysteine. Since the NysA-KS^S^-S413N mutant retained some activity, it was further proposed that acetyl-CoA can be used as starter unit too. Although this residue is not present in RimA, we cannot rule out the possibility that acetyl-CoA and butyryl-CoA could also be loaded directly by RimA for the biosynthesis of **2a** and **4a** respectively.

The remaining 13 Type I PKS elongation modules are responsible for the correct formation of the polyketide chain for **2a** and **4a** biosynthesis. Acetate would be incorporated as elongation unit in all modules by decarboxylative condensation of malonyl-CoA, except modules 7 and 13, which would incorporate propionate and butyrate by decarboxylative condensation of methylmalonyl-CoA or ethylmalonyl-CoA units, respectively. The incorporation of methylmalonyl-CoA and ethylmalonyl-CoA to the growing polyketide yields methyl and ethyl side chains, respectively on the macrolactone ring.

Several routes have been proposed for providing the methylmalonyl-CoA precursor for polyketide biosynthesis: (i) the isomerization of succinyl-CoA, catalyzed by the coenzyme B_12_-dependent methylmalonyl-CoA mutase (MCM); (ii) carboxylation of propionyl-CoA, catalyzed by the propionyl-CoA carboxylase (PCC); (iii) methylmalonyl-CoA ligase (MatB) [26]. In the case of ethylmalonyl-CoA, two pathways have been described: catabolism of valine and the butyryl-CoA pathway [27]. Crotonyl-CoA reductase (CCR) was described as the key enzyme in the butyryl-CoA pathway, catalyzing the last step in the conversion of two acetyl-CoA molecules into butyryl-CoA. Besides the hydrogenation of crotonyl-CoA to butyryl-CoA, it has been recently reported that CCR also catalyzes the reductive carboxylation of crotonyl-CoA to ethylmalonyl-CoA, this last reaction being the physiologically favored reaction [28,29]. This reaction catalyzed by CCR is the key step in the recently discovered ethylmalonyl-CoA pathway, involved in assimilation of acetate in several bacterial species [28] and in the biosynthesis of different polyketides [30,31].

A crotonyl-CoA carboxylase/reductase encoded by *rimJ*, previously described as a crotonyl-CoA reductase, was found within the **4a** and **2a** gene cluster [20]. Interestingly, disruption of *rimJ* did not abolish **4a** production and, in addition, a new compound with a typical tetraene spectrum was detected along with **2a** [20]. At that time we hypothesized that *rimJ* mutant might unbalance the incorporation of starter or/and extender units by the loading module RimA or elongation step 13. In this work we aimed at a deeper characterization of the profile of polyene being produced by a *rimJ* mutant with reduced availability of some metabolic building blocks for polyene production. The versatility of RimA of *S*. *diastaticus* var. 108 towards starter units encouraged this approach for isolating recombinant strains producing different chemical structures.

We recently described the isolation of the *pcsA* gene, unlinked to the polyene biosynthetic cluster in *S*. *diastaticus* var. 108. This gene encodes a polyene carboxamide synthase, PcsA, involved in tailoring of the exocyclic carboxyl group of **2a** and **4a** into their carboxamide derivatives [32]. PcsA shows glutamine amidotransferase activity and belongs to the asparagine synthases B (Class II amidotransferases) [33,34], which recognizes the final polyenes **2a** and **4a** as substrates. Moreover, PcsA can also convert *in vivo* and *in vitro* not only **2a** and **4a** into their corresponding amides, but also pimaricin (a heterologous substrate) [17], revealing interestingly reduced selectivity of this enzymatic activity for polyene macrolides. A similar gene, *pcsB*, encoding another polyene carboxamide synthase, PcsB, but specific for converting pimaricin into its corresponding amide (AB-400), was recently reported [35]. Polyene amide derivatives showed higher antimicrobial activity than their parent compounds without increasing the haemolytic rate, suggesting increased selective toxicity against fungal membranes. Thus, use of this tailoring gene with reduced substrate selectivity within a genetic background, such as *S*. *diastaticus* var. 108, makes this strain a promising system for the attempted generation of new molecules with improved biological activity.

## Material and Methods

### Bacterial strains, cloning vectors and growth conditions

Bacterial strains and plasmids are described in Table 1. *S*. *diastaticus* var. 108 and its engineered derivatives were cultured in SYM2 medium [36] for tetraene production analysis, and liquid TSB (Oxoid) for plasmid and total DNA extraction. *Streptomyces lividans* TK21 [37] was used as a general cloning host and grown on solid R5 medium and in liquid YEME medium [37]. *E*. *coli* JM101 [38] was grown on Luria-Bertani (LB) agar or in LB broth [39]. *P*. *chrysogenum*, *C*. *krusei*, *A*. *niger*, *C*. *albicans* and *F*. *neoformans*, used for testing antifungal activity, were grown in RPMI-1640 medium (Sigma, catalog. No N-3503) supplemented with 0.165M MOPS (morpholinepropanesulfonic acid) buffer. The pH was adjusted to 6.9–7. pGAe-1 [16], pIJ2925 [37], PM1 phage [40], pHJL401 [41], pIJ4090 [37] and pIJ922 [42] vectors were used for cloning.

**Table 1:** Bacterial strains and plasmids used in this study.

Strain or plasmid	Properties	Reference
*S*. *diastaticus* var. 108	Wild-type (WT); CE-108 and rimocidin producer.	[19]
*S*. *diastaticus* var. 108/PM1-709B	WT derivative with *rimJ* disrupted by integration of PM1-709B.	This work
*S*. *diastaticus* var. 108::PM1-709B/860	WT derivative with *rimJ* disrupted by integration of PM1-709B and transformed with pSM860.	This work
*S*. *diastaticus* var. 108/780	WT derivative by transformation with pSM780. CE-108 and rimocidin producer.	This work
*S*. *diastaticus* var. 108/781	WT derivative by transformation with pSM781. Rimocidin producer as majority polyene.	This work
*S*. *lividans* TK21	General cloning host	[37]
*S*. *lividans* TK21/pSM858	*S*. *lividans* TK21 WT derivative transformed with pSM858 plasmid	[32]
*E*. *coli* JM101	General cloning host	[38]
*Penicillium chrysogenum* ATCC10003	Antifungal activity assays	ATCC
*Aspergillus niger* ATCC1004	Antifungal activity assays	ATCC
*Issatchenkia orientalis* CECT 1688	Antifungal activity assays	CECT
*Filobasidiella neoformans* CECT 1078	Antifungal activity assays	CECT
PM1-709B	1.8 kb *Xho*I-*Bgl*II fragment from pGAe-1 [16] carrying the *ermE* gene and 840 bp *BamH*I-*Sac*I internal fragment of *rimJ* cloned into the *Xho*I/*Sac*I sites of PM1 phage [40].	This work
pSM859	*Hind*III-*EcoR*I fragment from pSM858 (carrying *oriT* and *pcsA* under the control of *ermE*_P_*) cloned into the *Hind*III/*EcoR*I sites of pIJ2925 [37].	This work
pSM860	*Bgl*II-*EcoR*I fragment from pSM859 (carrying *ori*T and *pcsA* under the control of *ermE*_P_*) cloned into the *BamH*I/*EcoR*I sites of pIJ922 [42].	This work
pSM780	pHJL401 [41] derived vector carrying the *ermE*_P_* promoter from pIJ4090 [37] and *oriT*.	[32]
pSM781	17,866–16,005 bp fragment from the sequence deposited under accession number AY442225 isolated as *BamH*I present in the oligonucleotide CCR-D (CGGGATCCCGCCTTTTCCGGAGGC, 17849–17866 bp from AY442225) and *Ecl136*II of the chromosome cloned into the *BamH*I-*Ecl136*II sites of pSM780. This plasmid carries *rimJ* under the control of the *ermE*_P_* promoter.	This work

*ermE*_P_*: constitutive erythromycin-resistance promoter where the asterisk signifies the presence of a one-base-pair mutation [43].

### Genetic procedures

*E*. *coli* JM101 was grown and transformed as described elsewhere [39]. *Streptomyces* strains were manipulated as previously described [37]. Intraspecific conjugation was carried out as previously described [16]. DNA manipulations were performed as described by Maniatis *et al*. [39].

### Assay for tetraene production

0.2 ml of total culture was extracted with 0.8 ml of methanol. The extracts were filtered and 20 μl were applied to an HPLC with a Waters 600S Controller, equipped with a Waters 996 PDA. The chromatographic parameters and the mobile phases were: 2 min with 100% of B (ammonium acetate 20 mM pH 5, ethanol 20%), 2 min of a binary gradient up to 50% of A (methanol) and 50% of B (curve 6); 6 min of a binary gradient up to 100 of A (curve 9), and a constant flow of 0.7 ml/min. The chromatograms were monitored at a wavelength of 304 nm.

### HPLC-MS Assays

The mass spectra were determined in a 1100MSD HPLC connected to a quadrupole Agilent Technology Detector using electrospray as source and a positive ionization mode. The chromatographic conditions were the same as described above and the applied voltage was 150V.

### Spectroscopic and spectrometric measurements

UV/vis spectra were recorded on a Perkin-Elmer Lambda 15 UV/vis spectrometer. NMR spectra were measured on a Varian Inova 600 (600.7 MHz) spectrometer. ESIMS was recorded on a Finnigan LCQ with a Rheos 4000 (Flux Instrument) quaternary pump. HRMS was recorded by ESI MS on an Apex IV 7 Tesla Fourier-Transform Ion Cyclotron Resonance Mass Spectrometer (Bruker Daltonics, Billerica, MA, USA). Reserpine (MW 608) and leucine enkephalin (MW 555) were used as standards in positive and negative mode.

### Purification of polyenes

**2a**, **2b**, **4a** and **4b** were purified as previously described [16] by a similar protocol as described below for **3a** and **3b**. **1** was purchased from Sigma (catalog. No N-3503).

**3a** was purified from *Streptomyces diastaticus* var. 108::PM1-709B which was grown on solid SYM2 medium supplemented with erythromycin (25 *μ*g/ml) for the selection of chromosomal insertions. Four plates of 24 x 24 cm were used for purification and the yield was up to 40 mg of tetraene-containing sample per plate. After 6 days, the whole solid medium was fragmented through a syringe, extracted with four volumes of methanol and 25 mM formic acid, stirred for 1 hr and centrifuged at 5000 x g for 20 min to remove solid particles. The clear supernatant was concentrated by rotaevaporation to 10–20 x 10^6^ U/μl measured at a wavelength of 304 nm. The sample was stored in 80% methanol/water until use. The methanol-extracted samples were brought to 20% methanol with water and filtered to remove precipitated material. An Omnifit column (250 x 25 mm, Supelco Catalog No. 56010) packed with SP-Sepharose Fast Flow (GE Healthcare) was equilibrated in the same solution. **3a** and the rest of the carboxylated polyenes were eluted with the flowthrough whereas minority polyene amides were completely retained in the column. A mixture containing **3a** was applied to a semipreparative column (Supelcosil PLC-8, 250.0 x 21.2 mm). The chromatographic parameters and the mobile phases, controlled with a Waters Automated Gradients Controller, were: 12 min with 100% of B (ammonium acetate 20 mM pH 5, ethanol 20%), 43 min of a binary gradient up to 50% of A (methanol) and 50% of B (curve 6); 35 min of a binary gradient up to 100 of A (curve 8), and a constant flow of 5 ml/min. Fractions were collected at regular intervals (5 ml per fraction) and those carrying the purified compounds were pooled and subjected to an additional desalting step, as above, and finally freeze-dried twice.

**3b** was purified from *S*. *diastaticus* var. 108::PM1-709B/860 which was grown on solid SYM2 medium supplemented with erythromycin (25 *μ*g/ml) and thiostrepton (50 *μ*g/ml) for the selection of chromosomal insertions and plasmid markers, respectively. The solid medium was processed as described above. An Omnifit column (250 x 25 mm, Supelco Catalog No. 56010) packed with SP-Sepharose Fast Flow (GE Healthcare) was used as described for **3a** compound. **3b** and the rest of the polyene amides were retained in the column, which was exhaustively washed with the same solution and the polyene amides were eluted with 300 mM ammonium acetate pH 5 in 20% methanol. The fractions containing the mixtures of polyene amides were desalted and **3b** was separated by using a semipreparative column (Supelcosil PLC-8, 250.0 x 21.2 mm) as described above. **3b** compound was pooled, subjected to an additional desalting step and finally freeze-dried twice.

**3a**: UV absorbing, pale yellow solid (6.3 mg). **NMR data** see Table 2 and S1–S5 Figs **(+)-ESI MS**: *m/z* (%) = 726 ([M+H]^+^, 100). **(-)-ESI MS**: *m/z* (%) = 724 ([M-H]^-^, 100). (+)-**ESI HRMS**: *m/z* = 726.36981 [M+H]^+^, (calcd 726.37006 for C_36_H_56_NO_14_).

**Table 2:** ^1^H NMR and ^13^C NMR Data of 3a and 3b in DMSO-*d*_6_.

	3a		3b	
Position	^1^H (Int., mult, *J* [Hz]) ^a)^	^13^C ^b)^	^1^H (Int., mult., *J* [Hz]) ^c)^	^13^C ^b)^
1	-	173.0	-	173.0
2	**2.20 (1H, m)**	47.0	2.20 (1H, m)	47.0
2-Me	**1.08 (3H, d, 10.8)**	13.2	1.08 (3H, d, 7.0)	13.1
3	4.03 (1H, m)	67.7	4.03 (1H, m)	67.8
4	2.36, 2.28 (2H, m)	48.1	2.36, 2.30 (2H, m)	48.1
5	-	208.5	-	208.6
6	2.39, 2.24 (2H, m)	43.1	2.43, 2.24 (2H, m)	43.1
7	**1.49, 1.26 (2H, m) 2.06**	19.3	1.53, 1.28 (2H, m)	19.3
8	1.28, 1.20 (2H, m)	37.4	1.28, 1.20 (2H, m)	37.4
9	3.98 (1H, m)	67.6	3.98 (1H, m)	67.6
10	1.48 (2H, m)	45.6	1.45 (2H, m)	45.6
11	-	96.9	-	96.9
12	1.82, 1.11 (2H, m)	44.4	**1.89, 1.12 (2H, m)**	44.7
13	**4.00 (1H, m)**	65.4	4.02 (1H, m)	64.7
14	1.84 (1H, m)	57.8	1.92 (1H, t, 10.3)	56.6
CONH_2_	-	176.4	-	174.2
15	4.16 (1H, t, 8.4)	65.4	4.17 (1H, t, 9.6)	65.2
16	2.16, 1.53 (2H, m)	36.7	2.06, 1.51 (2H, m)	36.6
17	4.38 (1H, m)	74.1	4.37 (1H, m)	74.4
18	5.89 (1H, dd, 15.2, 8.2)	136.4	5.87 (1H, dd, 15.3, 8.4)	136.3
19	6.06 (1H, dd, 15.2, 10.7)	128.5	6.06 (1H, m)	128.5
20	6.32 (1H, m)	132.2	6.31(1H, dd, 13.9, 10.7)	132.9
21	6.13 (1H, m)	131.5	6.13 (1H, m)	131.2
22	6.13 (1H, m)	131.9	6.13 (1H, m)	131.9
23	6.13 (1H, m)	131.7	6.13 (1H, m)	131.8
24	6.11 (1H, m)	133.0	6.13 (1H, m)	133.2
25	5.60 (1H, m)	130.4	5.61 (1H, m)	130.4
26	2.39, 2.24 (2H, m)	39.0	2.41, 2.29 (2H, m)	39.0
27	4.88 (1H, m)	69.5	4.88 (1H, m)	69.5
28	1.17 (3H, d, 6.1)	20.2	1.16 (3H, d, 6.7)	20.2
Sugar				
1'	4.53 (1H, s)	95.9	4.39 (1H, s)	96.4
2'	3.75 (1H, d, 1.7)	68.0	3.69 (1H, d, 1.6)	68.5
3'	2.81 (1H, d, 4.7)	56.0	2.62 (1H, m)	56.0
4'	3.16 (1H, dd, 9.6, 8.9)	70.1	3.06 (1H, m)	70.9
5'	3.24 (1H, m)	72.7	3.12 (1H, m)	72.9
6'	1.17 (3H, d, 6.1)	17.8	1.16 (3H, d, 6.7)	17.8
OH/NH	7.18, 5.20 (brs)	-	7.32, 6.83 (brs)	-

^a)^ 300 MHz;

^b)^ 125 MHz;

^c)^ 600 MHz. Chemical shifts (δ) is expressed in ppm.

**3b**: UV absorbing, pale yellow solid (8.2 mg). UV/Vis (0.1 mg/ml MeOH): λ_max_ (log ε) = 317 (4.09), 302 (4.14), 287 (4.10) nm. **NMR data** see Table 2 and S6 Fig. **(-)-ESI MS**: *m/z* (%) = 769 ([M+HCOO]^-^, 100). **–ESIHR MS**: *m/z* = 725.38606 [M+H]^+^, (calcd 725.38605 for C_36_H_57_N_2_O_13_).

### Antifungal susceptibility testing

MICs were determined according to NCCLS document M27-A [44] and M38-P [45] for yeast and conidia-forming filamentous fungi, respectively. Due to the nature of the assayed compounds, the dilutions were made according to the indications for insoluble antibiotics in water. The transmittance of the cultures was adjusted with 0.82% NaCl up to values between 75–80% for *Filobasidiella neoformans* and *Issatchenkia orientalis*, 78–82% for *Aspergillus* and 74–76% for *Penicillium*. Each suspension was diluted in RPMI-1640 medium 1:100 for *Aspergillus* and *Penicillium*, and 1:2000 for *F*. *neoformans* and *I*. *orientalis*. **1**, **1a** and **4b** were dissolved in 2 g/L DMSO and, **2a**, **2b** and **3a** in 6 g/L DMSO. Each drug was serially diluted twofold in RPMI-1640 medium and the appropriate dilutions were finally diluted 1:50. The assays were performed in multi-well plates by mixing 50 μl of the polyene dilutions with 50 μl of the cellular suspension. MICs were interpreted after 24 hours incubation at 37°C for *Issatchenkia orientalis* and *Aspergillus niger* and 48 hours for *Filobasidiella neoformans* and *Penicillium chrysogenum*. For all the polyenes assayed, the endpoint was defined at the lowest concentration that completely inhibited growth. The polyenes used in the study were purified as described above.

### Hemolytic activity assay

The different polyenes were dissolved in DMSO at 5 nmol/*μ*l for **2b** and **4b**, 20 nmol/*μ*l for **3b**, and 0.05 nmol/*μ*l for **1**. Increasing quantities of the different polyenes were brought to a final volume of 25 μl of DMSO and mixed by gently shaking with 125 μl of PBS buffer containing 2.5% of horse blood. After incubation at 37°C for 30 min without agitation, cells were pelleted by centrifugation. The hemolysis was evaluated by measuring the absorbance at 545 nm. The values corresponding to total hemolysis were estimated with a suspension of 2.5% horse blood in distiller water. Horse blood was from Oxoid (defibrinated blood).

## Results

### Production of a new CE-108 derivative, CE-108D (3a), by genetic engineering

According to our previous findings [20], disruption of the *rimJ* gene, encoding a crotonyl-CoA carboxylase/reductase belonging to the rimocidin/CE-108 gene cluster, caused some interesting differences in the chromatographic profile of the produced polyenes compared to the wild type strain. The observed chromatographic profile of polyene production is consistent with the putative role of *rimJ* in controlling the cellular balance of ethylmalonyl-CoA (or possibly ethylmalonyl-CoA and butyryl-CoA) needed as starter unit for rimocidin production. Bearing in mind that ethylmalonyl-CoA is also needed for elongation step 13 in rimocidin/CE-108 biosynthesis, an altered intracellular concentration of ethylmalonyl-CoA units might be targeting two biosynthetic steps of the rimocidin/CE-108 pathway: the loading process and elongation step 13.

**Fig 2:**
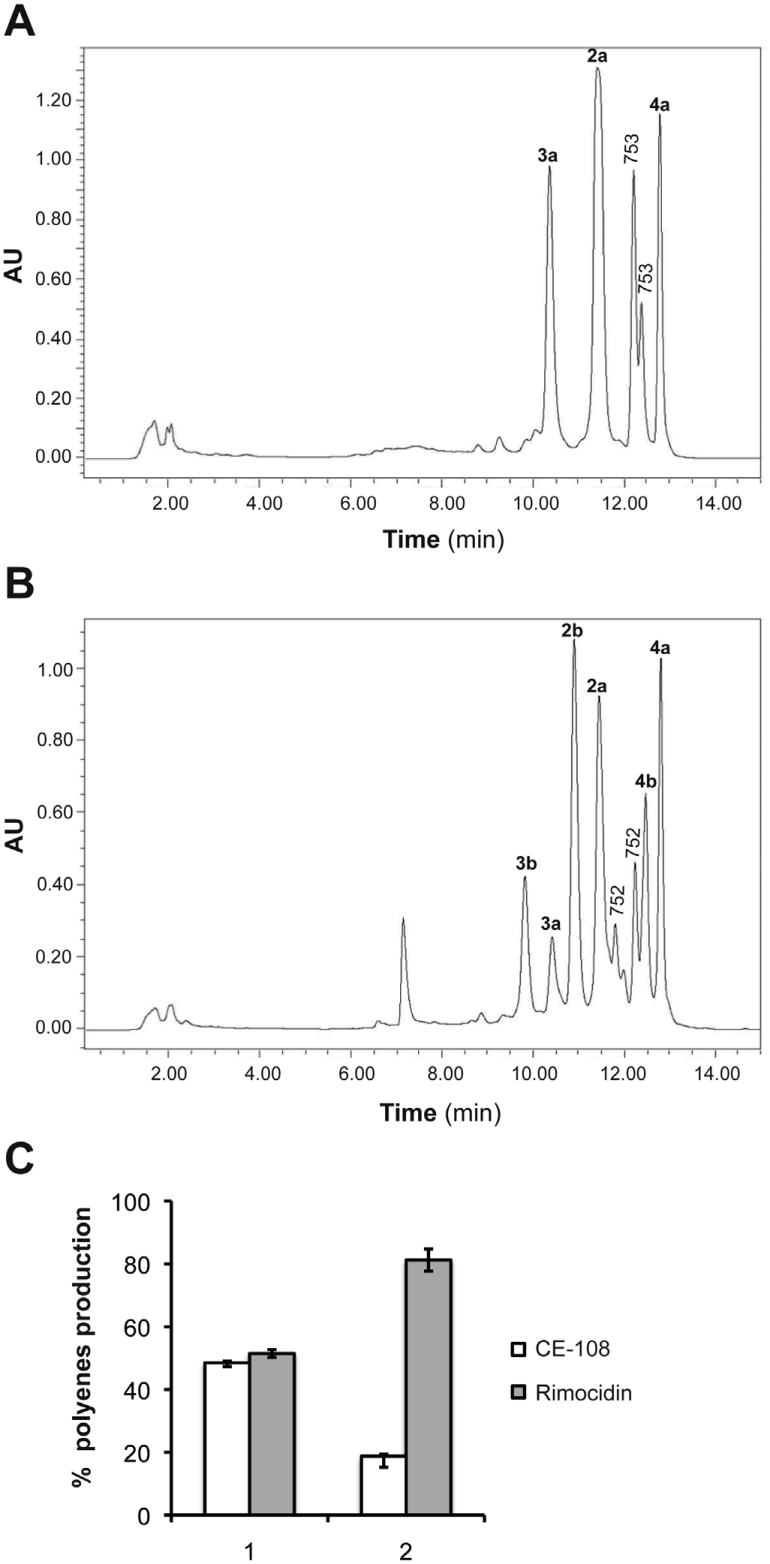
*rimJ* recombinants. (A) HPLC analysis of the fermentation broth of *S*. *diastaticus* var. 108/PM1-709B (*rimJ* disruptant). (B) HPLC analysis of the fermentation broth of the *rimJ* disruptant carrying *pcsA* under the control of the constitutive promoter *ermE*_P_* (*S*. *diastaticus* var. 108::PM1-709B/860); the numbers on the peaks in **A** and **B** correspond to the polyenes shown in Fig 1. Peaks marked with 753 and 752 are cited in the text. (C) Percentage of CE-108 and rimocidin production. Polyenes production was measured from liquid cultures by HPLC as described in Material and Methods. The data shown are the mean of three independent experiments. The standard deviation of the mean is indicated by error bars. **1**, WT control *S*. *diastaticus* var. 108/780 carrying the empty vector; **2**, WT derivative carrying *rimJ* under the control of the *ermE*_P_* promoter.

To further explore the *in vivo* specificity of PcsA toward the new compounds, we first had to design a new *rimJ* disruptant, as it shared the same thiostrepton resistance marker with the PcsA expression plasmid. We therefore exchanged the thiostrepton ressistance marker for erythromycin, which subsequently allowed the transformation with the PcsA expression plasmid (as discussed later). Thus, the *ermE* gene and an internal fragment of *rimJ* (positions 16,691 to 17,530 bp from the previously determined sequence, Accession Number AY442225) were cloned together into the *Xho*I/*Sac*I sites of the actinophage PM1 [40], replacing the thiostrepton resistance gene and part of the hygromycin resistance gene, as described in Table 1. The resulting recombinant phage carrying the erythromycin resistance gene, named PM1-709B, was used to infect *S*. *diastaticus* var. 108. The correct *rimJ* disruptant (*S*. *diastaticus* var. 108::PM1-709B) was confirmed by Southern blotting. HPLC analysis of the fermentation broth of this recombinant showed a clear decrease in **4a** production, an increase in **2a** and the overproduction of new compounds with typical tetraene spectra (Fig 2A). One of them, with a retention time lower than that of **2a** was called **3a** and two other compounds, with a retention time between **2a** and **4a** were additionally found, but were not fully elucidated. HPLC-MS analysis of these compounds revealed preliminary masses of 725 Dalton for **3a** and 753 Dalton for both new overproduced compounds (marked as 753 in Fig 2A). The loss of 14 units in **3a** compared with **2a** and the same loss for the other two compounds with respect to **4a** is consistent with the incorporation of an alternative unit by the loading module RimA and/or the extender module 13. The two compounds with a deduced mass of 753 Dalton could not be efficiently separated by HPLC and were thus not further investigated. Hence, we focused on the characterization of **3a**.

Also, we isolated *rimJ* and expressed it under the control of a constitutive promoter in the wild type strain. A DNA fragment from the *rim* cluster containing *rimJ* and the 5´end of *rimK* (17,866–16,005 bp of the sequence at accession number AY442225) and flanked by *Bam*HI/*Ehe*I sites, was fused with the *ermE*p* promoter [43] in the pHJL401 vector by several steps detailed in Table 1 (see also Material and Methods). This recombinant plasmid, named pSM781, carrying extra copies of recombinant *rimJ*, was introduced by transformation into *S*. *diastaticus* var. 108 to give rise to *S*. *diastaticus* var. 108/781. The presence of the plasmid was confirmed by direct plasmid extraction. The fermentation broth of this recombinant strain was tested for tetraene production by HPLC analysis and compared to the wild type control (*S*. *diastaticus* var. 108/780); the chromatograms showed that the production of **2a** was substantially reduced compared with the wild type in favour of an increase of **4a** (Fig 2C). This result confirms that the ethylmalonyl-CoA precursor required as starter and extender units (module 13) acts as a limiting factor in **4a** production by the wild type strain.

### 3a is a new substrate for the carboxamide synthase PcsA

In our previous report [32], PcsA was shown to exhibit a broad substrate specificity and converts the exoxyclic carboxyl group of **2a**, **4a** and pimaricin into the corresponding amides **2b**, **4b** and AB-400, respectively. Because the carboxamide polyenes were improved in some pharmacological properties, we were interested in knowing if the polyene derivative **3a** isolated from the *rimJ* mutant strain can be also be recognized by PcsA, and if so, if the carboxamide derivative has improved antimicrobial properties.

To answer these questions, the engineered tetraene **3a** was purified as described in Material and Methods and used as substrate for *in vitro* amidotransferase assays performed using cell-free extracts from *S*. *lividans* TK21/pSM858 as previously described [32]. The reaction products were analyzed by HPLC. A clear conversion of **3a** into another peak was observed (data not shown). The identity of the new peak in the amidation reaction was confirmed by HPLC-MS analysis, with an experimental mass for the reaction product of 724 Dalton for the putative amide of **3a**. The loss of 1 unit in the mass of **3a** strongly suggested that conversion of a carboxyl group into the amide took place, indicating that the new carboxyl polyene **3a** is a new substrate of PcsA activity. The new polyene amide was called **3b**.

### Suitably engineered *S*. *diastaticus* var. 108 overproduces the new carboxylated and amidated tetraene

The first step towards the characterization of **3a** and its corresponding amide **3b** was to generate a strain able to produce these new polyenes. For this purpose, a double recombinant strain was generated. Protoplasts of the lysogen *S*. *diastaticus* var. 108/PM1-709B (*rimJ* disruptant) were transformed with a construct carrying *pcsA* under the control of *ermE*_*P*_ * in the low copy number plasmid pSM860 (Table 1). Several colonies were selected with erythromycin and thiostrepton. The correct *rimJ* disruption and the presence of the plasmid pSM860 were both confirmed by Southern blotting and direct plasmid extraction, respectively.

As expected, HPLC analysis of the fermentation broth of the double recombinant *S*. *diastaticus* var. 108::PM1-709B/pSM860 clearly revealed overproduction of the expected polyene amide **3b** (Fig 2B). Other new peaks with retention times between **2a** and **4b** with masses of 752 Dalton deduced by HPLC-MS analysis could be the respective amides of the 753 Dalton carboxylated polyenes described above.

### Characterization of the new tetraenes

**3a** and **3b** were isolated from the recombinant strains *S*. *diastaticus* var. 108::PM1-709B and *S*. *diastaticus* var. 108::PM1-709B/pSM860, respectively, as described in Material and Methods.

#### Chemical structure elucidation of 3a and 3b

Both polyene macrolides were faint yellow solids. Compound **3a** was sparingly soluble in methanol and readily soluble in DMSO and pyridine, while **3b** was well soluble in methanol and DMSO.

The HR-ESI mass spectrum of the pale yellow powdery **3a** showed a *pseudo*molecular ion peak at *m/z* = 726.36927 [M+H]^+^, which corresponds to the ion formula C_36_H_56_NO_14_, and fits only with structure **3a** amongst the alternatives listed above. The ^1^H NMR spectrum showed very close similarity to those of **2a**, **2b**, **4a**, **4b**, CE-108C, and rimocidin C [16,17,19].

The ^1^H NMR spectrum displayed four signals in the *sp* ^*2*^ region at *δ* 6.32 (1H, m), between *δ* 6.05–6.15 (5H, m), at *δ* 5.89 (1H, dd), and at *δ* 5.65 (1H, m), with integration of eight protons in total. Two exchangeable signals appeared at *δ* 7.18 and 5.20 as broad singlets. One oxygenated methine at *δ* 4.53 was due to an anomeric proton, as the HSQC spectrum displayed. The other protons of the sugar moiety appeared in the range of *δ* 4.62–3.25; a methine group at *δ* 2.81 was possibly connected to a nitrogen atom. In the aliphatic region between *δ* 2.60–1.40, the spectrum showed a complex multiplet pattern, in addition to three methyl doublets: two at *δ* 1.17 and the third at *δ* 1.08 were present (S1 Fig).

In the ^13^C NMR spectrum, 36 carbon signals were observed, which is in agreement with the HR-ESI mass spectrum. These carbon signals could be classified as three carbonyls: one ketone CO at *δ* 208.5, two CO signals corresponding to an acid, amid or ester at *δ* 173.0 and 176.4. Eight *sp* ^*2*^ carbon signals were in the range of *δ* 136.4–128.4, two anomeric carbons gave signals at *δ* 96.9 (C_q_) and *δ* 95.9 (CH). Oxygenated carbons were observed between *δ* 74.1 to 65.5, which were attributed to C-3, 9, 13, 15, 17, 27, 2', 4' and 5'. Finally three methyls were present at *δ* 20.2, 17.8, and 13.2, respectively (S2 Fig).

To confirm the structure of **3a**, it was subjected to 2D NMR measurements. The H,H COSY spectrum showed a correlation series beginning with the methine carbinol at *δ* 4.38 assigned to H-17, which was coupled with one of the methylene protons at *δ* 2.16 assigned to H-16 and with the *sp* ^*2*^ methine doublet of doublets at *δ* 5.89 (H-18). The latter proton correlated with H-19 at *δ* 6.06, which in turn coupled with another *sp* ^*2*^ methine proton H-20 at *δ* 6.32. The signal at *δ* 2.81 (H-3') was correlated to the proton at *δ* 3.75 (H-2') and also with the methine proton at *δ* 3.16 (H-4'), which correlated to another methine at *δ* 3.24 to construct a part of the sugar moiety. The methylene protons at *δ* 2.15 and 1.53 assigned to H-16 coupled with the methine carbinol at *δ* 4.16 (H-15) (see Table 2; S3 and S4 Fig). The COSY and HMBC correlation confirmed the southern hemisphere and the structure of the amino sugar clearly. The anomeric proton H-1' (4.53) exhibited a ^3^ *J* correlation with C-17 (74.1), which confirmed the position of the sugar.

The interpretation of couplings in the northern part of the molecule was difficult, due to strong signal overlapping. The HMBC ^*3*^ *J* coupling of the proton at *δ* 4.88 (C-27) to the carbonyl at *δ* 173.0 (C-1) confirmed the lactone. The methyl protons of 2-Me displayed ^*3*^ *J* couplings with the lactone carbonyl, the carbinol carbon C-3 (67.7) and a ^2^ *J* coupling with C-2 (47.0). The methyl protons (2-Me) showed an additional COSY correlation with the methine at *δ* 2.20 (H-2), which in turn correlated to the carbinol methine at *δ* 4.03 (H-3). The HMBC correlations (S5 and S6 Figs) confirmed those relations observed in the COSY spectrum, which led to the complete elucidation of the structure of **3a** as shown in Fig 1. It should be mentioned, however, that there were no COSY or HMBC correlation visible between CH_2_-8 and CH_2_-9, and also not between the C-14-carboxyl group and H-13 or H-15, respectively, in both **3a** and **3b**.

Compound **3b** was obtained as a pale yellow powder and showed a typical tetraene UV spectrum with λ_max_ at 317, 302 and 287 nm similar to that of **3a**. The combined data of HR ESI MS (*m/z* = 725.38543 [M+H]^+^), ^13^C NMR and ^1^H NMR data (see Table 2) of **3b** delivered the molecular formula C_36_H_56_N_2_O_13_. The ^1^H NMR spectrum was very similar to that of **3a**. By comparing the molecular formula of **3b** and **3a**, the former one must have an amide group (CONH_2_) instead of the carboxylic group (COOH) in the latter one. The 2D NMR experiments showed the same correlations like **3a**, so that the structure **3b** is fully confirmed (Fig 1).

#### Antifungal properties of the recombinant polyenes

MICs were measured for **3a**, **3b**, and the previously characterized compounds **2a**, **2b**, **4a** and **4b**. Amphotericin B (**1**) was also included for reference. Broth microdilution MICs were determined according to NCCLS document M27-A [44] for *F*. *neoformans*, *C*. *krusei* and according to document M38-P [45] for *A*. *niger* and *P*. *chrysogenum* as described in Material and Methods.

**Fig 3:**
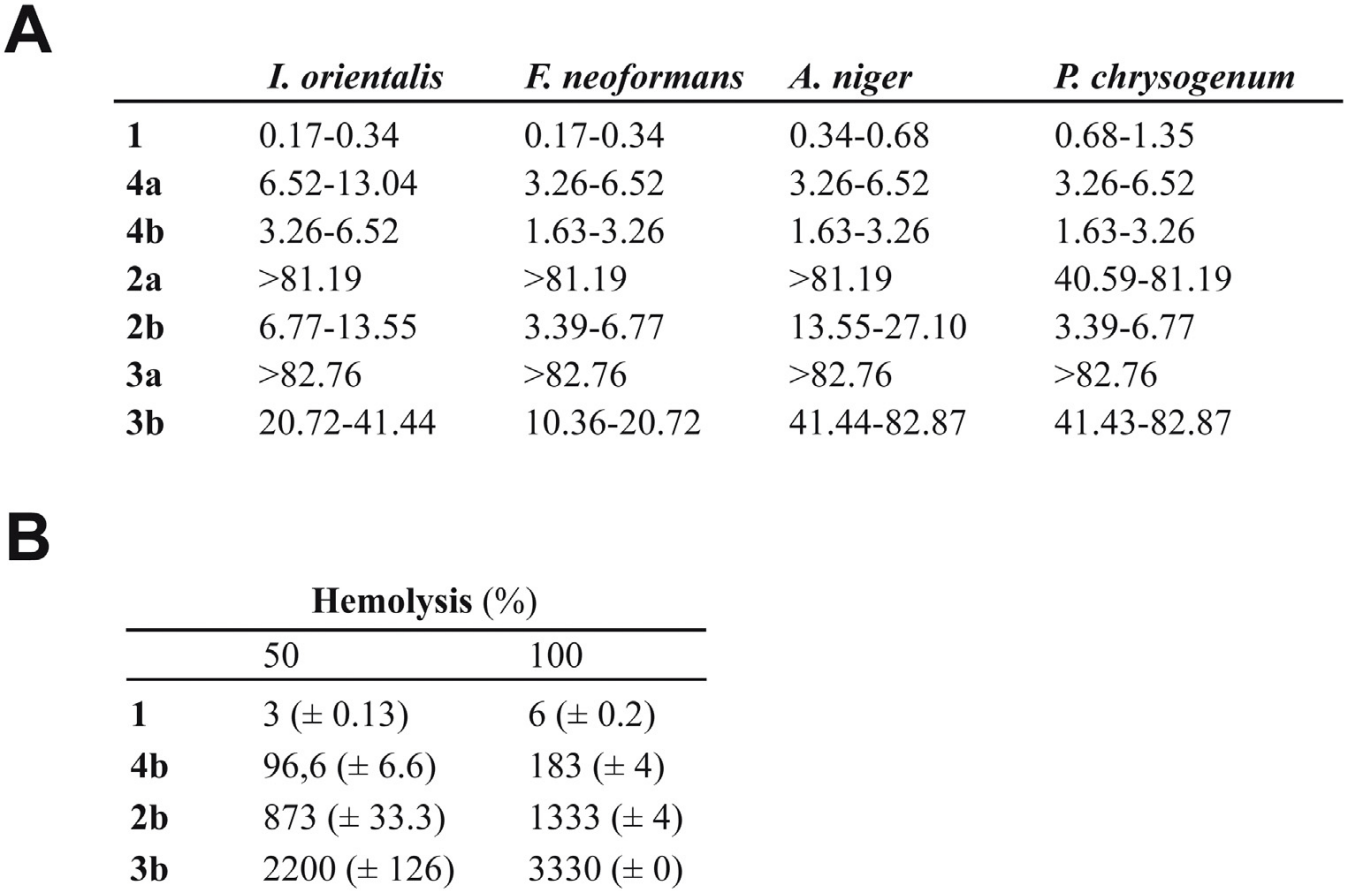
Biological activities for the polyenes tested. (A) *In vitro* susceptibilities of *Issatchenkia orientalis*, *Filobasidiella neoformans*, *Aspergillus niger* and *Penicillium chrysogenum* to seven antifungal agents. Minimal and maximum values of the range of concentrations for the MICs (μM) are detailed. Minimal values correspond to the lowest concentration of polyene in which growth appeared and maxima values were the lowest concentration that completely inhibited growth. Data are shown at 24 hours for *I*. *orientalis* and *A*. *niger* and at 48 hours for *F*. *neoformans* and *P*. *chrysogenum*. (B) Hemolytic activity of **1**, **4b**, **2b** and **3b**. Concentration in *μ*M of the polyenes for 50% and 100% hemolysis is expressed. The standard deviation is shown in parentheses.

The results revealed a low biological activity for the carboxylated polyene **3a** against all the microorganisms tested. The conversion of the side chain carboxyl group of **3a** into an amide group present in **3b** led to an increasing biological activity against all fungi tested (Fig 3A). As expected from previous data [16], the MIC values for the polyene amides **2b** and **4b** are substantially lower than those of the corresponding carboxyl polyene compounds against all fungi tested (Fig 3A).

For the amide derivatives, we observed that the biological activity of **3b** is lower than that of **2b**. We conclude from this that the substitution of an ethyl group at C-2 by a methyl residue led to a reduction in biological activity. In this sense, **4a** is more bioactive than **2a** and **3a** because it presents a lateral ethyl chain at C-2 besides a propyl chain at C-27 instead of a methyl group present in both **2a** and **3a**. So, interestingly, a decrease of biological activity occurs when the number of carbons in side chains at C-2 or C-27 is reduced.

#### Toxicity assays

Horse erythrocytes were used as a cellular model for this study since similar hemolytic activities have been reported for horse and human blood [16]. Since we had previously found that conversion of the free carboxyl group into an amide did not alter hemolytic activity, we confined the toxicity assays to the polyene amides due to their major antifungal activity. The hemolytic activity assays of **3b** were evaluated versus **1**, **2b** and **4b** as described in Material and Methods. Concentrations expressed in *μ*M of the different tetraenes producing 50% and 100% of hemolysis are detailed in Fig 3B. Maximum toxicity was found for **1**. It is noteworthy that, as occurred for the antifungal activity, the toxicity of the polyene amides assayed was directly related to the number of carbons in the side chains at C-2 and C-27, with toxicity being higher for the polyenes having longer side chains.

**Fig 4:**
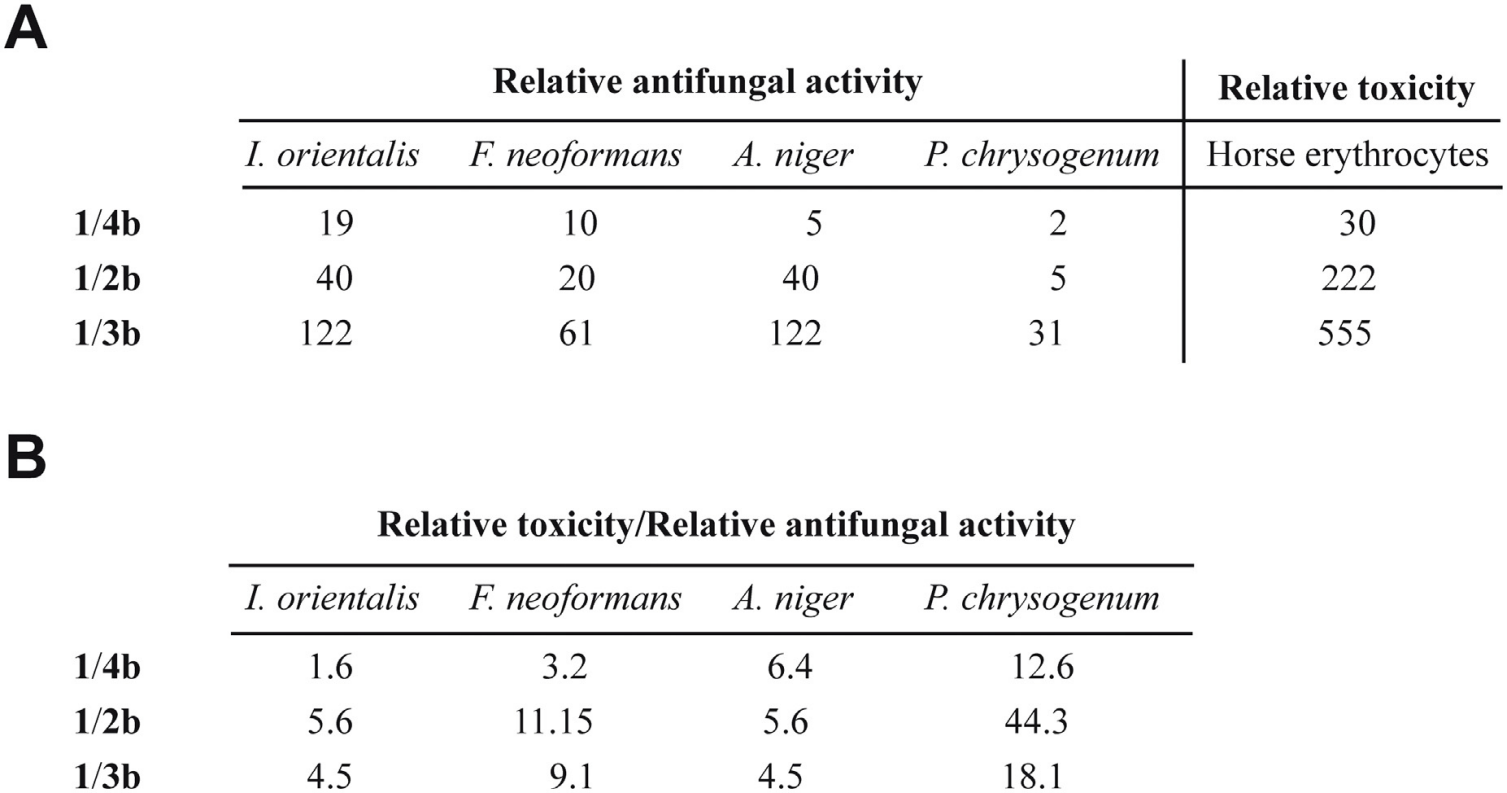
Relative antifungal activity and toxicity. (A) Number of times that **1** is more active against all the fungi tested and toxic in terms of hemolytic activity than the polyenes **4b**, **2b** and **3b**. (B) Ratio between relative toxicity and relative antifungal activities. The values indicate the times that the polyene is less toxic than **1** for the same level of inhibition against the fungi tested.

To correlate the antifungal activity and toxicity of these new compounds, Fig 4A shows the number of times that **1** is more active in terms of both antifungal activity and toxicity than the assayed polyenes. On a comparable level of inhibition against the tested fungi, all analyzed polyene amides showed lower toxicity than **1** (Fig 4B).

## Discussion

Here we report the *in vivo* biosynthesis of new rimocidin analogues through inactivation of the gene *rimJ*, which encodes a predicted crotonyl-CoA carboxylase/reductase in the rimocidin biosynthetic gene cluster in *S*. *diastaticus* var. 108. The newly produced macrolide antibiotics were furthermore shown to be substrates for the heterologously expressed carboxamide synthase PscA, which allowed the *in vivo* and *in vitro* generation of further rimocidin derivatives with increased bioactivity.

**Fig 5:**
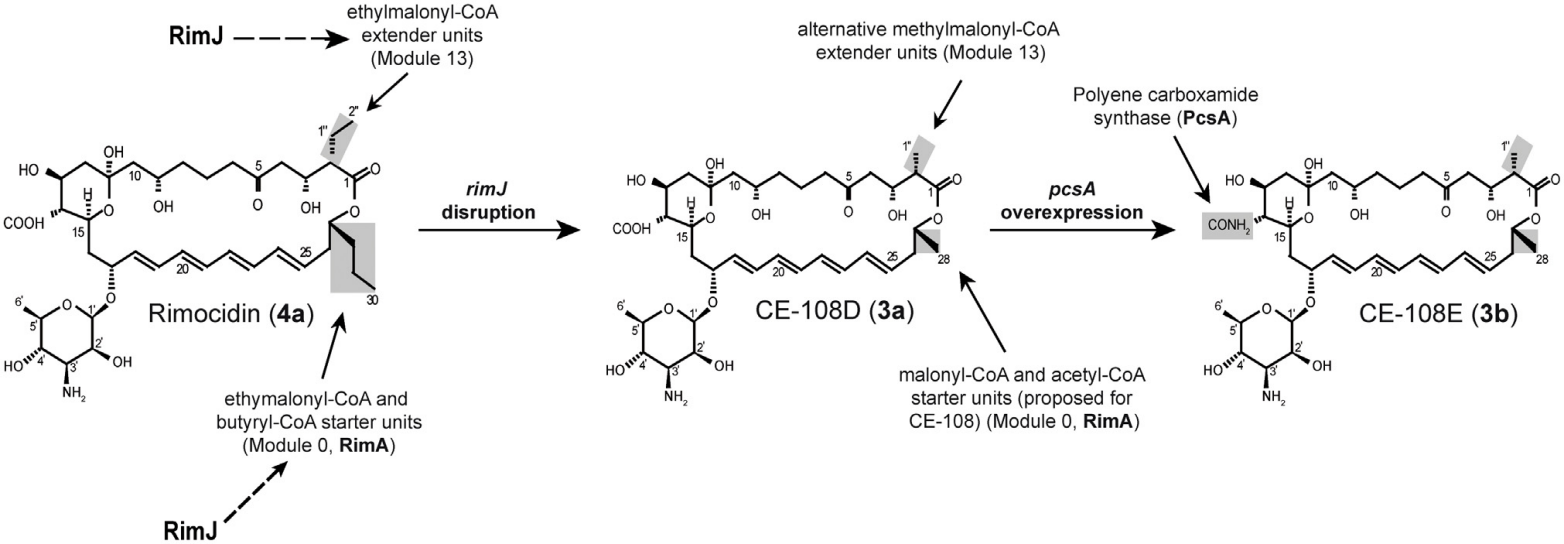
Proposed model for 3a and 3b biosynthesis.

**3a** and **3b** are structural derivatives of **2a** and **2b**, respectively, by substitution of an ethyl side chain with a methyl side chain at C-2. During **3a** formation, the elongation module 13 incorporates acetate (derived from methylmalonyl-CoA) rather than the naturally observed butyrate (derived from ethylmalonyl) in the biosynthesis of **2a** and **4a** [20], indicating a reduced selectivity for the PKS involved in elongation module 13, which can incorporate methylmalonyl-CoA units when the availability of ethylmalonyl-CoA units is reduced as a consequence of *rimJ* disruption. Once the polyketide chain is completed and cyclized, two tailoring reactions occur: the conversion of the side chain methyl group at C-14 into a free carboxyl group by cytochrome P450 monooxygenase RimG; and the incorporation of mycosamine at C-17 by glycosyltransferase RimE [20]. The carboxylated compound **3a** is the product of this biosynthetic pathway but can be a substrate for the polyene carboxamide synthase PcsA activity, as we have confirmed here through *in vitro* amidation assays, giving rise to **3b** (Fig 5).

Besides this new characterized compound, other uncharacterized peaks with typical tetraene spectra were found in the fermentation broth of the *rimJ* recombinant whose deduced masses of 753 Dalton in both cases are in accordance with rimocidin (**4a**) derivatives carrying: (i) an ethyl side chain at C-27 by incorporation of methylmalonyl-CoA, or possibly methylmalonyl-CoA and propionyl-CoA, in the loading module; (ii) a methyl side chain at C-2 by incorporation of methylmalonyl-CoA as extender unit in elongation module 13 as described above for **3a** biosynthesis. Thus, we expect the presence of both rimocidin derivatives in this recombinant strain and probably a new derivative carrying both chemical modifications.

As we report here, the availability of ethylmalonyl-CoA units constitutes a limiting factor in **4a** production, so that all the other carboxylated tetraene derivatives produced by this strain are a consequence of a low availability of these precursors. The presence of **4a** and **2a** in the fermentation broth of the *rimJ* mutant suggests the presence of at least one other CCR activity related to primary metabolism in *S*. *diastaticus* var. 108; the corresponding gene and others related to valine catabolism also involved in formation of these carboxylic acids [46] could be good tools for trying to overproduce new interesting compounds by gene disruption. An appropriated genetic manipulation of the producer strain *S*. *diastaticus* var. 108, with a highly flexible PKS, would favor the overproduction of these new carboxylated tetraenes.

Loading module RimA could have a higher flexibility than described for **2a** and **4a** recognizing not only acetyl-CoA and butyryl-CoA but probably propionyl-CoA, making it a good tool for obtaining polyketide derivatives by combinatorial biosynthesis. PimS0, the loading module for pimaricin biosynthesis, has a high degree of identity with RimA (70%), but PimS0 cannot incorporate either propionyl-CoA or butyryl-CoA as starter units in the same genetic context as RimA [47].

The new carboxylated compound **3a** turned out to be a new substrate for PcsA activity, converting it into its amide **3b**. However, as occurred for **4a** and **2a**, **3a** was not recognized by the polyene carboxamide synthase PcsB involved in the conversion of pimaricin to its corresponding amide [35]. The improved pharmacological properties of the polyene amide **3b**) compared to the carboxylated compound **3a** suggests the interesting possibility of extending this chemical modification to other polyenes.

The major compounds produced by a strain are not necessarily the most interesting. It is worth seeking also minor components in the fermentation broth of the producer strain to obtain new metabolites with improved pharmacological properties. A new CE-108 derivative produced by incorporation of malonyl-CoA as alternative extender unit by elongation module 13 is also possible. HPLC-MS analysis confirmed the presence of other uncharacterized compounds with typical tetraene spectra and minor retention times in the fermentation broth of *S*. *diastaticus* var. 108. whose masses of 711 and 710 are consistent with this new derivative and its corresponding amide. Here we show a clear correlation between the number of carbons in the side chains at C-2 and C-27 and the antifungal activity, but the loss of antifungal activity when the number of carbons is reduced is compensated by a substantial reduction in toxicity (Fig 4). This suggests these compounds as potential alternatives to **1** in the treatment of systemic infections.

## Conclusions

In a previous study [32], we described a polyene carboxamide synthase, PcsA, from *S*. *diastaticus* var. 108 involved in a post-PKS activity converting the free carboxyl group widespread in most natural polyene macrolides into an amide group. PcsA not only converts the carboxyl group of **2a** and **4a** (the predominant polyenes produced by this strain) into their respective polyene amides **2b** and **4b**, but also the heterologous substrate pimaricin, leading to an increase in selectivity towards fungal membranes in all cases [16]. Importantly, the high flexibility of the PKS involved in polyene macrolide biosynthesis in this strain allowed the overproduction of new polyene derivatives by disrupting a gene encoding a crotonyl-CoA carboxylase/reductase (*rimJ*) in the polyene biosynthetic gene cluster. One of them, **3a**, obtained by incorporation of an alternative extender unit in elongation step 13 of the biosynthetic pathway, turned out to be a new substrate for PcsA. **3b**, the corresponding carboxamide derivative of **3a**, showed improved pharmacological properties with respect to the parental products. The reduced selectivity of this enzymatic activity plus the high flexibility of the *S*. *diastaticus* var. 108 PKS are useful tools for generating new antifungal drugs.

## Supporting Information

S1 Fig ^1^H NMR spectrum of CE-108D (3a) in DMSO-*d*_6_ at 300 MHz. (DOCX)

S2 Fig ^13^C NMR spectrum of CE-108D (3a) in DMSO-*d*_6_ at 125 MHz. (DOCX)

S3 Fig Selected correlations observed in the H,H COSY spectra of CE-108D (3a). (DOCX)

S4 Fig H,H COSY spectrum of CE-108D (3a) in DMSO-*d*_6_ at 600 MHz. (DOCX)

S5 Fig Selected HMBC correlations of CE-108D (3a). (DOCX)

S6 Fig H,H COSY NMR spectrum of CE-108E (3b) in DMSO-*d*_6_ at 600 MHz. (DOCX)
