# Supplementary figures and images for: New Rimocidin/CE-108 Derivatives Obtained by a Crotonyl-CoA Carboxylase/Reductase Gene Disruption in Streptomyces diastaticus var. 108: Substrates for the Polyene Carboxamide Synthase PcsA
Source: PLoS One. 2015 Aug 18;10(8):e0135891. doi: 10.1371/journal.pone.0135891 (PMC4540446; doi:10.1371/journal.pone.0135891)

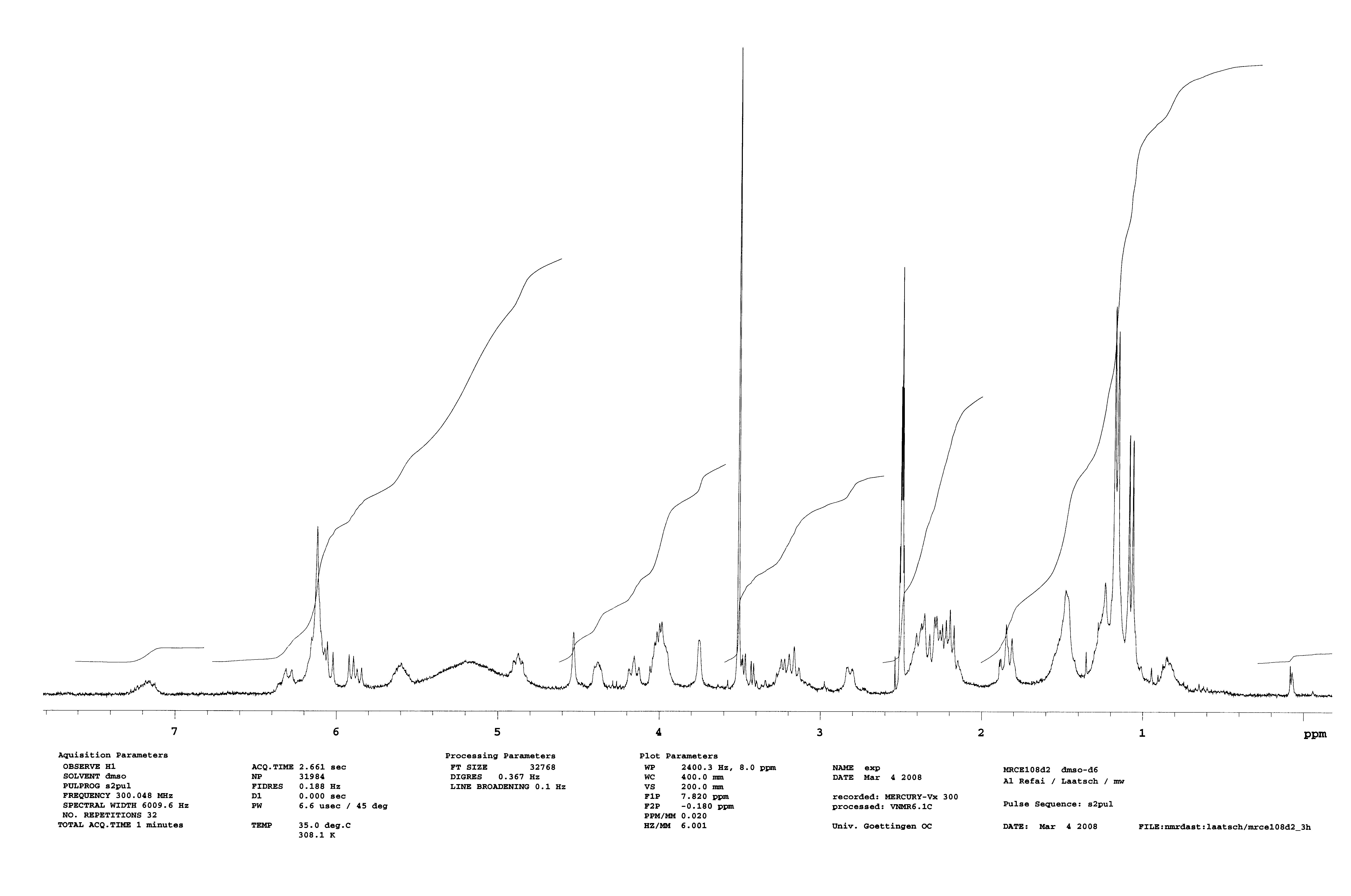


S1 Fig. 1H NMR spectrum of CE-108D (3a) in DMSO-*d*6 at 300 MHz

Supplement: S1 Fig — (DOCX) [file pone.0135891.s001.docx]

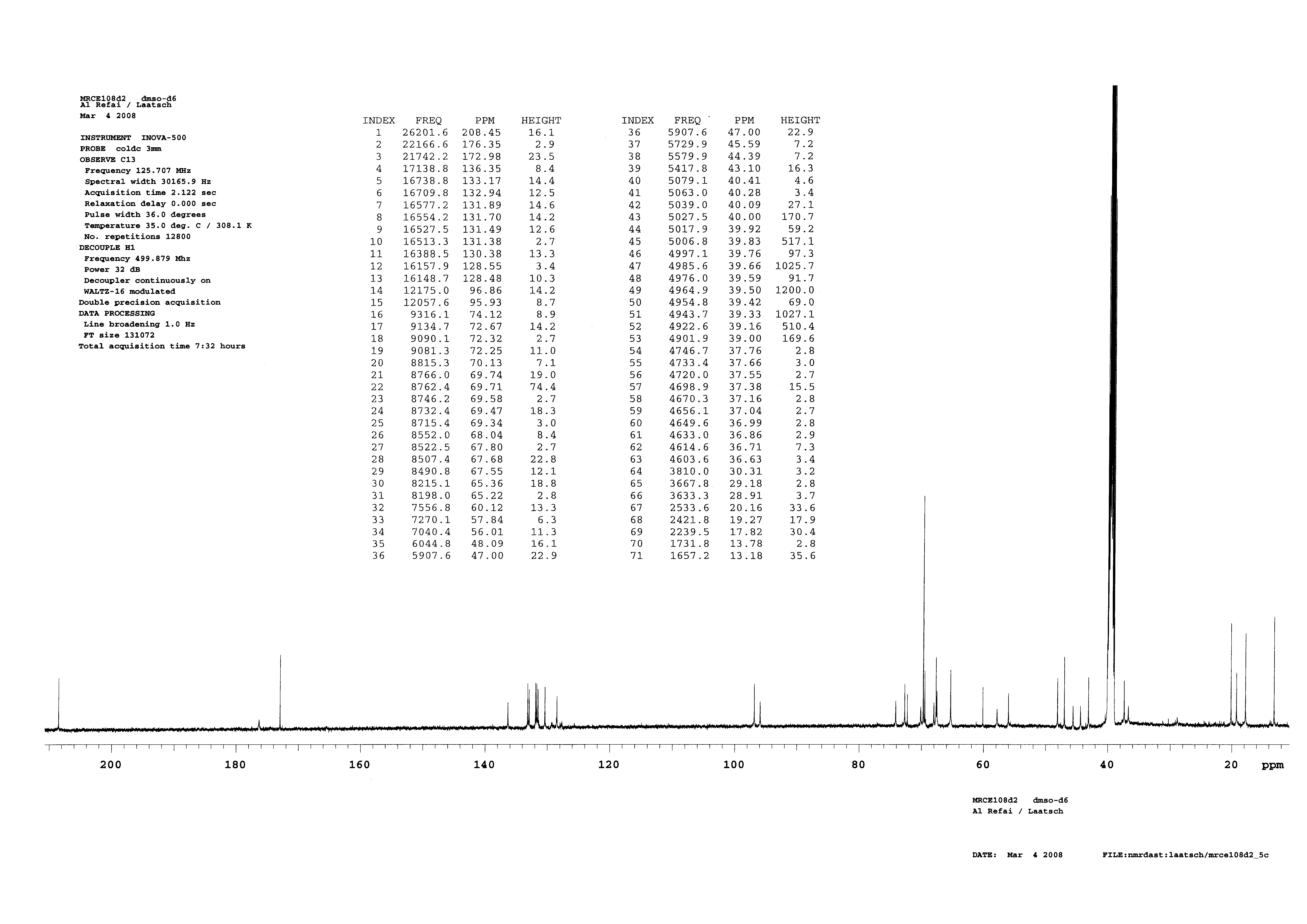


**S2 Fig.** 13C NMR spectrum of CE-108D (**3a**) in DMSO-*d*6 at 125 MHz

Supplement: S2 Fig — (DOCX) [file pone.0135891.s002.docx]

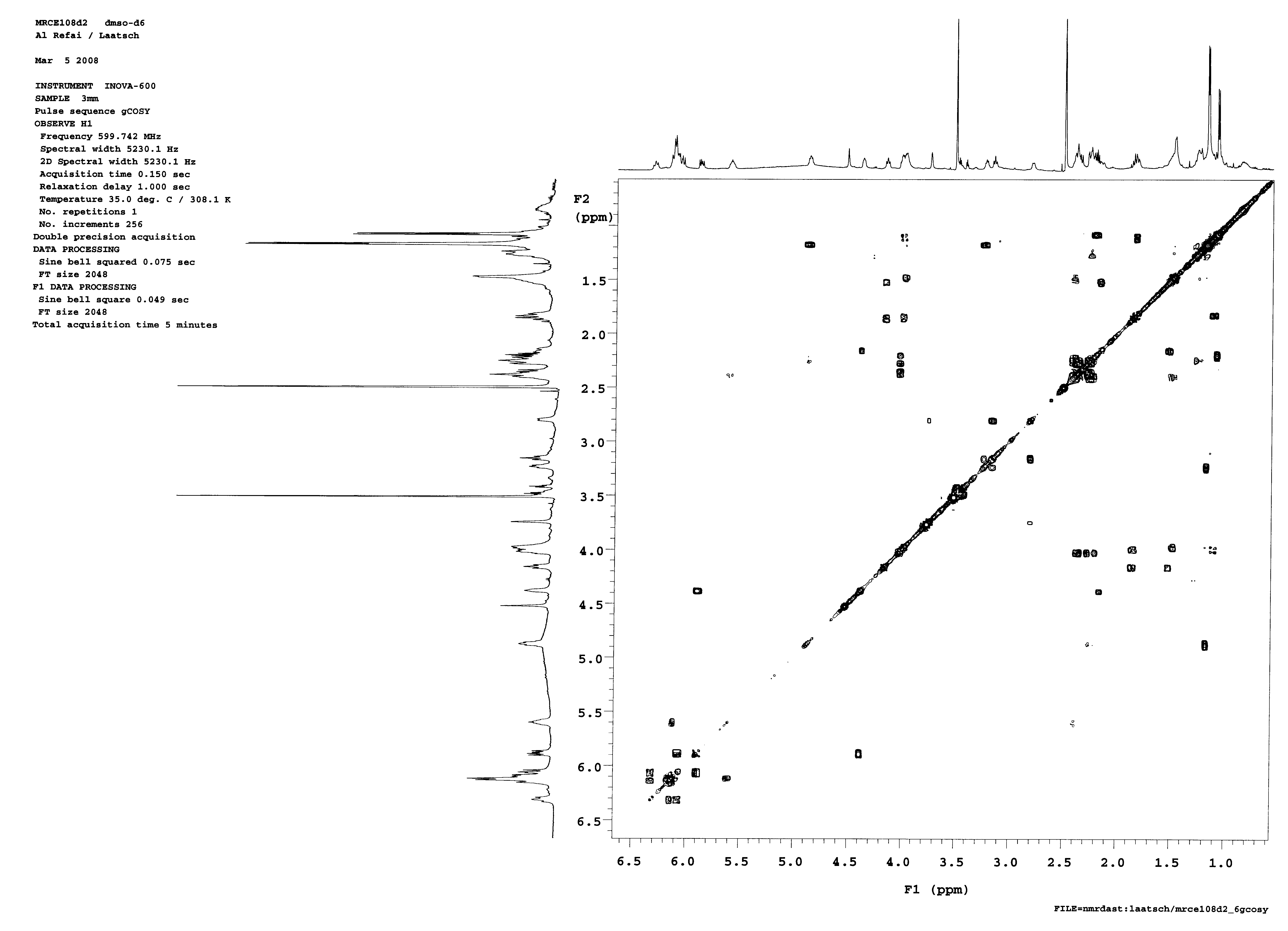


**S4 Fig.** H,H COSY spectrum of CE-108D (**3a**) in DMSO-*d*6 at 600 MHz

Supplement: S4 Fig — (DOCX) [file pone.0135891.s004.docx]

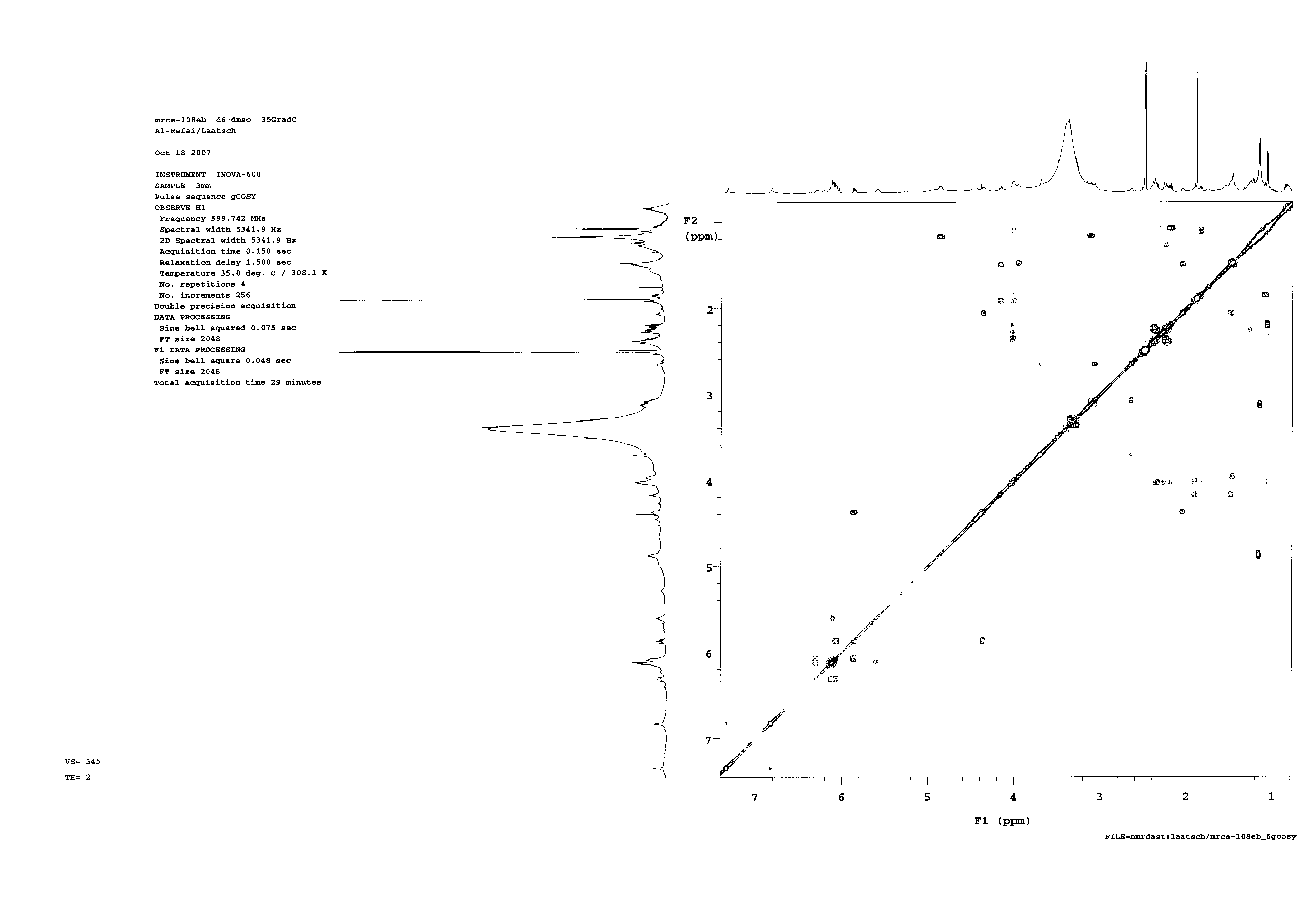


**S6 Fig.** H,H COSY NMR spectrum of CE-108E (**3b**) in DMSO-*d*6 at 600 MHz

Supplement: S6 Fig — (DOCX) [file pone.0135891.s006.docx]
