# Supplementary material for: New Rimocidin/CE-108 Derivatives Obtained by a Crotonyl-CoA Carboxylase/Reductase Gene Disruption in Streptomyces diastaticus var. 108: Substrates for the Polyene Carboxamide Synthase PcsA
Source: PLoS One. 2015 Aug 18;10(8):e0135891. doi: 10.1371/journal.pone.0135891 (PMC4540446; doi:10.1371/journal.pone.0135891)
Supplement: S3 Fig — (DOCX) [file pone.0135891.s003.docx]

S3 Fig. Selected correlations observed in the H,H COSY spectra of CE-108D (3a)
