# Supplementary material for: New Rimocidin/CE-108 Derivatives Obtained by a Crotonyl-CoA Carboxylase/Reductase Gene Disruption in Streptomyces diastaticus var. 108: Substrates for the Polyene Carboxamide Synthase PcsA
Source: PLoS One. 2015 Aug 18;10(8):e0135891. doi: 10.1371/journal.pone.0135891 (PMC4540446; doi:10.1371/journal.pone.0135891)
Supplement: S5 Fig — (DOCX) [file pone.0135891.s005.docx]

**S5 Fig.** Selected HMBC correlations of CE-108D (**3a**)
